# *Mycobacterium avium* subsp. *hominissuis* Infection in 2 Pet Dogs, Germany

**DOI:** 10.3201/eid1406.071463

**Published:** 2008-06

**Authors:** Verena Haist, Frauke Seehusen, Irmgard Moser, Helmut Hotzel, Ulrich Deschl, Wolfgang Baumgärtner, Peter Wohlsein

**Affiliations:** * University of Veterinary Medicine Hanover, Hanover, Germany; † Federal Research Institute for Animal Health, Jena, Germany; ‡ Boehringer Ingelheim Pharma GmbH & Co. KG, Biberach/Riss, Germany

**Keywords:** Canis familiaris, dog, mycobacteriosis, Mycobacterium avium-intracellulare complex, Mycobacterium avium subsp. Hominissuis, zoonoses, letter

**To the Editor:** The genus *Mycobacterium* contains various obligate and opportunistic pathogens of animals, which may also be transmitted to humans and cause disease in, thus exhibiting a considerable zoonotic potential (*1*,*2*). During the past few decades, members of the *Mycobacterium avium-intracellulare* complex (MAIC) emerged as pathogens of human diseases, including lymphadenitis in children, pulmonary tuberculosis-like disease, and disseminated infections (occurring predominantly in immunocompromised persons, particularly AIDS patients) (*1*,*2*). Similarly, important animal diseases are caused by members of this group, e.g., avian tuberculosis and paratuberculosis in ruminants (*1*). MAIC includes *M. intracellulare* and 4 subspecies of *M. avium*, namely, *M. avium* subsp. *avium*, *M. avium* subsp. *hominissuis*, *M. avium* subsp. *silvaticum*, and *M. avium* subsp. *paratuberculosis* (*3*,*4*). Whereas members of the *M. tuberculosis* complex are transmitted by direct host contact, MAIC species are acquired predominantly from environmental sources, including soil, water, dust, and feed. Subclinical infections are common among birds (*1*,*2*).

*M. avium* strains differ from *M. intracellulare* by containing the insertion sequence (IS) IS*1245* (*3*) and are further discriminated by terms of IS*901* (*4*). Avian isolates (*M. avium* subsp. *avium*) are usually positive for IS*901* and represent the main pathogen of avian tuberculosis (*5*). In contrast, mammalian isolates are IS*901*-negative and have been designated as *M. avium* subsp. *hominissuis* because of their predominant hosts. This subspecies is only weakly virulent for birds but causes disease in animals and humans (*5*).

Even though *M. tuberculosis* and *M. bovis* are the common etiologic agents of canine mycobacteriosis, dogs are reported to be relatively resistant to *M. avium* infection (*6*,*7*). Nonetheless, sporadic cases usually show nonspecific clinical signs, whereas necropsy consistently reveals granulomatous inflammation in numerous organs, including lymph nodes, intestine, spleen, liver, lung, bone marrow, and even spinal cord (*7*,*8*). The predominant involvement of the gastrointestinal tract indicates an oral route of infection (*7*,*8*), and simultaneously increases the risk for human infection by fecal spread of mycobacteria.

Our report concerns 2 young dogs, a 3-year-old miniature schnauzer and a 1-year-old Yorkshire terrier, that lived in different geographic regions in Germany. Both had had therapy-resistant fever, lethargy, progressive weight loss, and generalized lymphadenomegaly for several weeks and were euthanized after a final phase of diarrhea. Necropsy findings, similar in both dogs, included generalized enlargement of lymph nodes with a whitish, granular to greasy cut surface, leading to intraabdominal adhesions by extensive involvement of mesenteric lymph nodes. In the terrier, the greater omentum and a part of the right apical lung lobe showed changes similar to those in the lymph nodes. Furthermore, numerous white 1-mm nodules were found in the spleen (both dogs), liver (schnauzer) and costal pleura (terrier).

Histologic examination showed (pyo-)granulomatous inflammation of lymph nodes, tonsils, liver, spleen, and greater omentum. Additionally, pyogranulomatous pleuropneumonia was present in the terrier, and a granulomatous enteritis and pyelitis in the schnauzer. The granulomatous lesions frequently exhibited central necrosis surrounded by macrophages, epitheloid cells, and few neutrophils (Figure, **panel A**). However, multinucleated giant cells or mineralization was not observed. In both animals, Ziehl-Neelsen stain demonstrated large numbers of acid-fast bacilli within macrophages (Figure, **panel B**). Samples of lymph nodes and lung were processed for mycobacterial culture by using standard procedures (Löwenstein-Jensen, Stonebrink medium). Colonies emerging after 2-week incubation at 37°C were investigated by PCR targeting IS*1245* and IS*901* (*3*,*4*). In all samples, *M. avium* subsp. *hominissuis* was identified by growth characteristics as well as presence of an IS*1245*-specific and absence of an IS*901*-specific PCR product. Additionally, sequencing of *hsp65* was conducted (*9*), which indicated *M. avium* subsp. *hominissuis* in both dogs (GenBank accession nos. EU488724 and EU488725).

**Figure:**
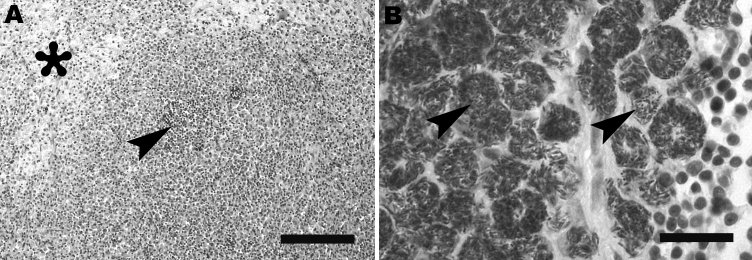
A) Mesenteric lymph node of Yorkshire Terrier shows diffuse granulomatous lymphadenitis with extensive infiltration of macrophages, foci of pyogranulomatous inflammation (arrowhead), and focal necrosis (asterisk). Hematoxylin and eosin stain; scale bar represents 100 μm. B) Retropharyngeal lymph node of schnauzer shows innumerable acid-fast bacilli (arrows) within the cytoplasm of macrophages. Ziehl-Neelsen stain; scale bar represents 25 μm.

Despite improved therapeutic approaches, MAIC infection represents a frequent bacterial complication in persons with AIDS. However, several studies showed a very low incidence of *M. avium* subsp. *avium* infections in humans. Thus, most of these HIV-related infections are attributed to *M. avium* subsp. *hominissuis* (*2*,*5*). Unfortunately, the subspecies of *M. avium* was not identified in most canine cases reported in the literature (*7*,*8*). Nonetheless, different serotypes of *M. avium*, corresponding to either *M. avium* subsp. *avium* or *M. avium* subsp. *hominissuis*, have been identified sporadically (*6*,*10*). The source and route of infection were unclear in all reports including ours, albeit repeatedly observed enteritis strongly suggested an oral mode of infection. A common environmental or wildlife reservoir represents the most probable source of *M. avium* infection for both humans and animals. However, there is also evidence of direct transmission (*1*–*3*). Therefore, *M. avium* subsp. *hominissuis* infection in dogs may comprise a considerable zoonotic potential, particularly if pet dogs with close contact to the owner are affected and if prolonged nonspecific clinical signs and intestinal involvement occur, as demonstrated here.
